# Beyond Words: Embracing Migration Percentage as the Universal Measurement for Hip Displacement in Children With Cerebral Palsy by Radiologists and Orthopedic Surgeons

**DOI:** 10.7759/cureus.48786

**Published:** 2023-11-14

**Authors:** Daniel Raftis, Sarah Dance, Laura Mazudie Ndjonko, Ahmed Elabd, Sean Tabaie

**Affiliations:** 1 Orthopaedic Surgery, The George Washington University School of Medicine and Health Sciences, Washington, USA; 2 Orthopaedic Surgery, Children’s National Hospital, Washington, USA; 3 Orthopaedic Surgery, Children's National Hospital, Washington, USA

**Keywords:** msk radiology, pediatric orthopedic surgery, migration percentage, hip dislocation, diagnostic imaging, hip displacement, cerebral palsy

## Abstract

Introduction: Migration percentage (MP) is the standard radiographic measurement to quantify hip displacement in cerebral palsy (CP) hip surveillance programs. We aim to evaluate the use of MP and other descriptors of hip displacement in radiographic impressions by radiologists and orthopedic surgeons before and after the introduction of hip surveillance guidelines at our institution.

Methods: CP patients who underwent hip surveillance imaging at our institution in 2016 were retrospectively identified, and their radiographic impressions were collected between 2016 and 2019. Only patients with radiology and orthopedic impressions for the same image were included. The inclusion of MP was documented and compared between the two groups before and after the hip guidelines were implemented in 2018. We also examined the use of other qualitative descriptors for hip displacement within the impressions. Fisher’s Exact test was used for statistical significance (p<0.05).

Results: Two hundred and fifty-one radiographs were analyzed. One radiology (0.40%) and 33 orthopedic impressions (13.15%) incorporated MP (p<0.001). No statistical significance was found between the inclusion of MP before and after 2018 in the radiology group. In contrast, the orthopedic group showed a significant increase in MP inclusion following guideline implementation, rising from 12 to 21 incidences (p=0.013). Descriptors for hip displacement were more commonly utilized than MP for both orthopedic surgeons and radiologists.

Conclusion: MP is underutilized in radiologic and orthopedic impressions in hip surveillance programs. Extensive education for both specialties regarding MP is crucial for the successful management of hip displacement in CP hip surveillance programs.

## Introduction

Hip displacement is the second most prevalent orthopedic comorbidity among children with cerebral palsy (CP). However, it often remains asymptomatic until the hip reaches a state of significant displacement [1,2]. To mitigate the risk of significant displacement, hip surveillance programs have been established for children with CP in Sweden and Australia, which have demonstrated their effectiveness in enhancing the early detection of hip displacement, subsequently enabling timely interventions to prevent distressing dislocations [2-4]. An essential component of these surveillance programs involves the standardization of radiographic assessments, with the migration percentage (MP), also known as the Reimers Migration Index, serving as the primary metric [5-7]. Numerous studies have shown the validity and reliability of MP in terms of intra- and inter-rater consistency, rendering it one of the most commonly adopted measures within well-established hip surveillance protocols [8,9].

In 2017, the American Academy for Cerebral Palsy and Developmental Medicine (AACPDM) published guidelines outlining the procedures for conducting hip surveillance among children with CP in the United States. These guidelines firmly advocated for the utilization of the migration percentage (MP) as the established radiographic measurement for quantifying hip displacement [https://www.aacpdm.org/publications/care-pathways/hip-surveillance-in-cerebral-palsy]. However, subsequent investigations have revealed a noteworthy pattern: MP is not a standard term within the vocabulary of general radiologists when they assess hip radiographs of children with CP within their respective institutions [10-12]. Rather, these studies have indicated that radiologists tend to employ a diverse array of terminologies to characterize hip displacement, frequently leading to variations from the impressions formed by orthopedic surgeons [10].

In 2018, our institution distributed the guidelines for hip surveillance, aiming to advance the early assessment and treatment of hip displacement among pediatric patients with cerebral palsy. However, an evaluation of this adoption has yet to be conducted. Therefore, the purpose of this study is to examine the presence of the migration percentage (MP) and other common terms used to describe hip displacement in radiographic interpretations, both before and after 2018. Additionally, a comparison of these interpretations was conducted between radiologists and orthopedic surgeons. Our hypothesis suggests that the incorporation of the MP has not been widely embraced by either radiologists or orthopedic surgeons.

## Materials and methods

Database and patient selection

An IRB-approved, retrospective study was performed using data from 2016-2019 at a single US-based academic institution. In 2018, a hip surveillance database was established at our institution that collects retrospective and prospective demographic, radiologic, and interventional information for all CP patients. Pediatric patients with CP who received imaging for routine monitoring of hip displacement in 2016 were identified from this database. Once these patients were identified, any radiographic impressions for hip and pelvis X-rays from 2016-2019 were collected from their electronic medical record (EMR). Only images that had both an associated radiologist impression and an orthopedic surgeon impression were included in our analysis. Radiology impressions were taken from the formal radiographic reports, whereas orthopedic surgery impressions were taken from the same day assessment and plan of the clinical note.

Demographics and qualitative analysis

Demographic information, including patient age, gender, and race, as well as CP category and gross motor function classification system (GMFCS) stages, were identified from the hip surveillance database. The percentage of each demographic category compared to the total population was calculated. Each radiographic impression was reviewed to determine the presence of specific descriptors, including "migration percentage"/"Reimers index", "Shenton's Line", "acetabular index/acetabular angle", "acetabular dysplasia", "subluxation", "dislocation", "coverage", and variations like "normal/good/well/nicely". A binary assessment was executed, entailing an assignment of one when the term was detected and 0 when it was absent, regardless of its frequency within the interpretation. In cases where none of these terms were discernible within the interpretation, a value of one was assigned to the "other" category. The cumulative frequency of each term was recorded for both orthopedic surgeons and radiologists, thereby facilitating the computation of the prevalence of each term.

Statistical analysis

The inclusion of MP was counted for both orthopedic surgeons and radiologists over the entire range from 2016 to 2019. Then, the prevalence was compared between both groups before and after 2018. Fisher's exact test was used to compare orthopedic surgeons with radiologists. This statistical method was chosen due to its utility in determining the significance of differences between groups or variables, especially with small sample sizes like our own. By calculating exact probabilities instead of relying on large sample approximations, Fisher analysis provided accurate and reliable statistical inferences, enhancing the validity of the results obtained by this study. We determined statistical significance as a p-value of less than 0.05.

## Results

Demographics

A total of 120 children with CP were identified from the hip surveillance database, all of whom underwent hip/pelvis radiographs in 2016. Of the 120, 100 patients had radiographs that included both orthopedic and radiologic impressions regarding hip displacement and were subsequently included in the analysis. From those patients, 251 radiographic impressions were collected over four years.

The average age was 8.28 years old (range: 2-17 years old). In terms of demographic distribution, 52% of the patients were male, while 48% were female. The largest racial group was black or African American, comprising 41% of the cohort, followed by Caucasians at 24%, Asians at 3%, and others at 29%. Of the patients included, 54% were classified as quadriplegic. Within the remaining subset, 21% were identified as diplegic, 5% as hemiplegic, 1% as triplegic, and 19% were categorized as having an unknown classification. An analysis of the Gross Motor Function Classification System (GMFCS) levels revealed that 30% were classified as GMFCS V, 8% as GMFCS IV, 6% as GMFCS III, 5% as GMFCS II, and 2% as GMFCS I, while 49% were not officially assigned a GMFCS stage (Table 1).

**Table 1:** Patient Demographics GMFCS = Gross Motor Function Classification Scale

Categories	Number
Total Patients	100
Total Radiographs	251
Average Age (Range)	8.28 (2-17 years old)
Gender	Males: 52%
	Females: 48%
Race	African American: 41%
	Caucasian: 24%
	Asian: 3%
	Others: 29%
Type of Cerebral Palsy	Quadriplegic: 54%
	Hemiplegic: 5%
	Triplegic: 1%
	Unknown: 19%
GMFCS level	I: 2%
	II: 6%
	III: 5%
	IV: 8%
	V: 30%
	Unknown: 49%

Inclusion of migration percentage

**Figure 1:**
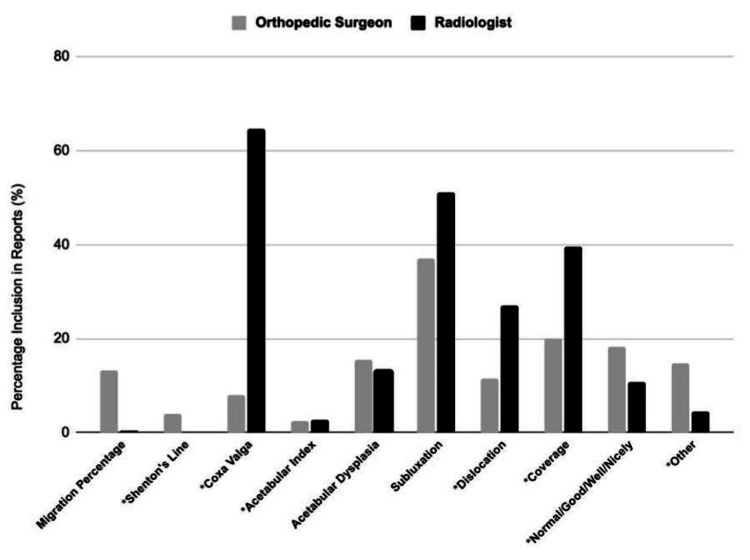
Frequency of Common Descriptors Used in Hip Surveillance X-ray Impressions Between Orthopedic Surgeons and Radiologists (*) denotes a significant finding (p<0.05)

**Figure 2:**
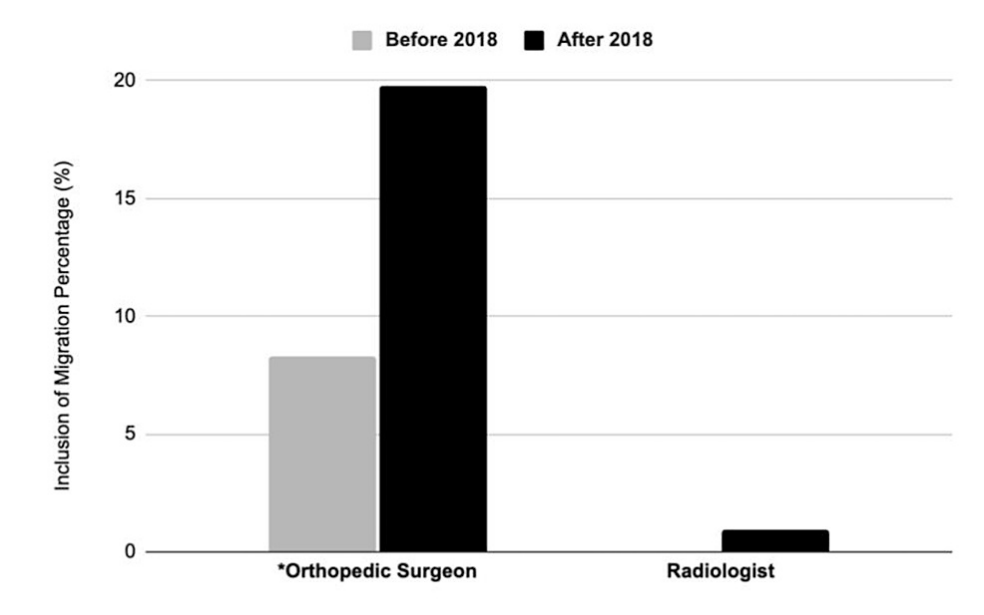
Percent Inclusion of Migration Percentage in Radiographic Impressions Before and After 2018 by Orthopedic Surgeons and Radiologists (*) denotes a significant finding (p<0.05)

Of the 251 radiographs ordered from 2016-2019, one (0.40%) radiologist impression included MP, compared to 33 (13.15%) orthopedic surgeon impressions (p<0.001) (Table 2, Figure 1). When comparing the inclusion of MP before and after 2018, radiologist impressions went from 0 (0%) to 1 (0.94%) (p = 0.874). The inclusion of MP in orthopedic surgeon impressions went from 12 (8.28%) before 2018 to 21 (19.81%) after 2018 (p=0.013) (Table 3, Figure 2).

**Table 2:** Migration Percentage and Other Common Descriptors From 2016-2019

Categories	Orthopedic Surgeons	Radiology	
	n (%)	n (%)	P-Value
Migration Percentage	33 (13.15)	1 (0.40)	<0.001
Shenton's Line	10 (3.98)	0 (0)	0.002
Coxa Valga	20 (7.97)	162 (64.54)	<0.001
Acetabular Index/Angle	6 (2.39)	7 (2.79)	1
Acetabular Dysplasia	39 (15.54)	33 (13.15)	0.52
Subluxation	93 (37.05)	128 (51.00)	0.002
Dislocation	29 (11.55)	68 (27.09)	<0.001
Coverage	50 (19.92)	99 (39.44)	<0.001
Normal/good/well/nicely	46 (18.33)	27 (10.76)	0.02
Other	37 (14.74)	11 (4.38)	<0.001

**Table 3:** Comparing the Inclusion of Migration Percentage Before and After 2018 Between Orthopedic Surgeons and Radiologists

Categories	Before 2018	After 2018	
	n (%)	n (%)	P-Value
Total Reports	145	106	
Orthopedic Surgeons	12 (8.28)	21 (19.81)	0.013
Radiologists	0 (0)	1 (0.94)	0.874

Other descriptors found in impressions

Quantifying other common descriptors found in radiographic impressions, orthopedic surgeons included the terms “Shenton’s Line” in 3.98%, “coxa valga” in 7.89%, “acetabular index/angle” in 2.39%, “acetabular dysplasia” in 15.54%, “subluxation” in 37.05%, “dislocation” in 11.55%, “coverage” in 19.92%, “normal/good/well/nicely” in 18.33%, and something other than all of these terms in 14.74% of impressions. In comparison, radiologists included the terms “Shenton’s Line” in 0%, “coxa valga” in 64.54%, “acetabular index/angle” in 2.79%, “acetabular dysplasia” in 13.15%, “subluxation” in 51.00%, “dislocation” in 27.09%, “coverage” in 39.44%, “normal/good/well/nicely” in 10.76%, and something other than all of these terms in 4.38% of impressions (Table 2, Figure 2).

## Discussion

The efficacy of successful hip surveillance programs relies significantly on the standardization of radiographic images to detect and monitor hip displacement in CP patients. Among the already established hip surveillance programs, the single most frequently cited barrier is inconsistent radiology reporting [13]. Although MP is the gold standard for measuring hip displacement in children with CP, as hypothesized, it has yet to be fully adopted by radiologists and orthopedic surgeons alike. Over the span of four years, merely 13.15% of radiographic interpretations for hip/pelvis radiographs in CP pediatric cases included the MP measurement in impressions by orthopedic surgeons, compared to a mere 0.4% by radiologists.

In lieu of the comprehensive adoption of MP as a definitive metric, our analysis reveals a diverse array of descriptors-such as "coverage" or "subluxation"-that are more frequently utilized when interpreting hip/pelvis radiographs, spanning both orthopedic surgeons and radiologists. Analyzing the impact of disseminating hip surveillance guidelines to both groups, our findings demonstrate that while the incorporation of MP did increase for orthopedic surgeons following the guidelines, the same upward trend was not observed among radiologists.

Several recent studies have assessed the extent to which the migration percentage (MP) is included in radiology reports. Siemens et al. conducted a study to determine the presence of MP in hip radiographs of children with CP at an institution lacking a formalized hip surveillance program [12]. Among the 92 radiographs examined by radiologists, only one report included MP. In contrast, Miller et al. investigated the incorporation of MP in a considerably larger facility with an established hip surveillance program [11]. They analyzed images from 960 patients spanning five years and found that 14.2% of radiographic interpretations included MP for bilateral hips, while 6.4% included it for a single hip. Our assessment of MP inclusion by radiologists yielded results similar to those of Siemens et al., even after our institution updated its hip surveillance guidelines in 2018. Miller et al.'s findings, though higher than ours, still indicated that MP is not consistently reported by radiologists in the context of hip surveillance radiographs.

Neither of these studies, however, undertook a comparison between radiology impressions and those of orthopedic surgeons. The Joint Commission on Accreditation of Healthcare Organizations mandates the dual interpretation of radiographs by both orthopedic surgeons and radiologists in clinical settings. Often, radiology interpretations are reported after patients have been seen by orthopedic surgeons [14]. Due to this, clinical decisions are commonly based on the surgeon's interpretation, which demonstrates the importance of measuring MP amongst surgeons for proper hip surveillance. Our findings indicate that orthopedic surgeons are more inclined to incorporate MP in their reports for the same radiographs, in contrast to radiologists.

Rather than incorporating the migration percentage (MP), a majority of radiographic interpretations included terminology describing the positioning of the patients' hips. In our study, it was observed that terms such as "acetabular dysplasia," "subluxation," "coverage," and variations like "normal/good/well/nicely" were more frequently included in orthopedic surgeon impressions than MP. Upon evaluating radiologist impressions, we found that all the measured descriptors were more commonly used, with "coxa valga" and "subluxation" appearing in over 50% of the impressions, implying that migration percentage is not a standardized radiographic measurement used by radiologists. The nuanced differences in what is more commonly utilized by radiologists and orthopedic surgeons may also highlight a difference in training when it comes to how each specialty reads hip and pelvis radiographs. Furthermore, MP is a radiographic measurement that orthopedic surgeons use to determine the timing of surgical hip interventions, which may contribute to its wider use among orthopedic surgeons compared to radiologists.

Both Miller et al. and Siemens et al. qualitatively assessed radiologic impressions, utilizing many of the same descriptors as our study. Siemens et al. attempted to correlate each descriptor term with their respective MP measurement and discovered an absence of consistent correspondence between descriptors and MP severity. For instance, the term "subluxation" was employed to describe radiographs with MP measurements ranging from 10%-130% [12]. Miller et al., on the other hand, found that terms like "description of coverage," "acetabular index," and "acetabular dysplasia" appeared in over half of the radiology reports [11]. Interestingly, these descriptors were not as frequently encountered in our analysis, revealing a lack of cross-institutional consistency in these qualitative descriptors. While these descriptors are not incorrect to include, they are not considered to be the gold standard for evaluating hips in this patient population.

The challenge with using these descriptors lies in their lack of specificity, rendering them inadequate in providing a clear grasp of disease severity due to their subjective nature. Our analysis underscores this issue, as distinct descriptors are employed by radiologists and orthopedic surgeons for the same radiographs. The overarching objective of hip surveillance is to systematically monitor patients to identify and address abnormalities before they can deteriorate. Additionally, the MP and its progression serve as crucial guidance in clinical decision-making, particularly in determining the optimal timing for surgical hip interventions [15,16]. The establishment of standardized measurements for tracking hip progression stands as a crucial element of effective surveillance, necessitating specific and easily replicable metrics such as the MP. While MP is necessary to properly evaluate CP hip displacement, it is also important to note the importance of including other more descriptive terms, such as “acetabular index” and “coxa valga,” in these radiographic reports as well. These and other similar terms add descriptive language that cannot be gleaned with the sole use of MP. While we advocate for the increased use of MP in radiographic reporting, we also realize that it cannot be the sole measurement used in the successful management of CP hip pathology.

The significance of education in standardizing the interpretation of radiographs in hip surveillance cannot be overstated. In 2018, our institution released guidelines for hip surveillance in children with CP. These guidelines were disseminated to all healthcare providers at our institution who treat CP patients, including orthopedic surgeons and radiologists. Upon comparing the utilization of the MP before and after the guideline implementation, a significant increase in MP inclusion was observed among orthopedic surgeons, while the same increase was not seen among radiologists. Despite the increase, the compliance rate for surgeons was only approximately 20% for radiographs, implying that both specialties have room for improvement.

A study conducted by Milks et al. pursued a similar objective, comparing MP inclusion before and after standardizing radiographic techniques and reporting practices among radiologists [10]. Much like our findings, their initial evaluation of 108 children showed no reported MP. However, five months following the implementation of an extensive educational program, 90% of radiology reports featured the inclusion of MP. In addition to disseminating an updated hip surveillance guideline to radiologists, the institution in the Milks et al. study imparted education through monthly newsletters, focused training sessions, and even a mandatory self-guided online training module [10]. Adopting a comparable level of educational resources to better equip both radiologists and orthopedic surgeons is pivotal in establishing successful hip surveillance programs within any institution.

This study is not without limitations. First, the radiographic impressions were derived from a single institution, thereby lacking generalizability. Second, unforeseen confounders may have contributed to our results, such as whether CP hip surveillance was included as an indication for hip/pelvis radiographs. As MP is used solely in the evaluation of CP hip displacement, not including that the patient has a diagnosis of CP in the indications may contribute to underreporting of MP among radiologists. Additionally, a limitation arises from the absence of documented patient positions during imaging, implying a likely lack of standardization in this aspect. Such variability in patient positioning could potentially contribute to the observed disparities in radiographic interpretations, as proper positioning is a critical factor in ensuring reliable MP measurements [17]. However, it's important to note that our study did not assess the accuracy of MP measurements, suggesting that correct positioning should not have changed whether physicians chose to include MP or not. In forthcoming endeavors, it will be imperative not only to provide comprehensive education regarding MP measurement but also to emphasize the importance of standardized patient positioning for optimal monitoring. Lastly, our research focused on assessing the inclusion of MP prior to and following the distribution of updated hip surveillance guidelines. Consequently, our study assumes that all radiologists and orthopedic surgeons received and had equal opportunities to review these guidelines. To address this, the implementation of mechanisms to monitor the adoption of standardized practices during ongoing education, such as the completion of online modules and random monthly reviews of radiographic impressions, could potentially aid in addressing this limitation.

## Conclusions

Our study supports the existing literature, underscoring that the migration percentage (MP) is not commonly integrated into radiology interpretations for assessing hip displacement, and this observation extends to orthopedic surgeons as well. Hip-surveillance programs have demonstrated their effectiveness in mitigating missed diagnoses and associated complications in various institutions worldwide. The integration of standardized MP measurement into radiographic interpretations stands as a pivotal element for the triumph of hip-surveillance initiatives. Consequently, dedicating substantial effort to comprehensively educate all radiologists and orthopedic surgeons engaged in the care of children with cerebral palsy about the significance of MP represents a crucial next stride for our institution and others aspiring to establish successful hip surveillance programs.
